# The impact of mother-infant rooming-in and continuous skin-to-skin contact on newborns

**DOI:** 10.3389/fped.2025.1577094

**Published:** 2025-04-24

**Authors:** Huaihui Chu, Jiqin Ye, Jie Chen, Jianhong Dang, Qiaozhen Lu, Lingling Li

**Affiliations:** ^1^ Department of Obstetrics, C.N. Maternity & Infant Health Hospital, Shanghai, China; ^2^ Department of Obstetrics and Gynecology, Second Affiliated Hospital of Naval Medical University, Shanghai, China; ^3^ Department of Neonatology, C.N. Maternity & Infant Health Hospital, Changning District, Shanghai, China

**Keywords:** mother-infant rooming-in, skin-to-skin contact, physiological weight loss in newborns, breastfeeding, neonatal jaundice

## Abstract

**Background:**

Mother-infant skin-to-skin contact (SSC) is a key component of Early Essential Newborn Care (EENC), recommended by the World Health Organization to improve neonatal outcomes. Despite its global adoption, SSC implementation in China remains inconsistent, with limited evidence on its impact in Chinese populations.

**Objective:**

To explore the effects of mother-infant rooming-in and continuous SSC on newborn weight, breastfeeding rates, and the use of medications for jaundice.

**Methods:**

A total of 2205 women who delivered at Shanghai Changning District Maternal and Child Health Hospital between January and December 2022, including a routine rooming-in care group (1120 cases) and an rooming-in with continuous SSC group (1,085 cases) retrospectively collected from medical records. Both groups of newborns received early essential neonatal care within 90 min of birth, followed by rooming-in with their mothers. Newborns in the routine rooming-in care group received routine rooming-in care, while those in the rooming-in with continuous SSC group were also subjected to continuous SSC with their mothers. The changes in newborn weight, breastfeeding rates, and the use of jaundice medications were compared between the two groups.

**Results:**

There was no statistically significant difference in the birth weight between the two groups (*P* > 0.05). The weight loss after birth, comparing 7% and 9% weight loss, was lower in the rooming-in with continuous SSC group than in the routine rooming-in care group (*P* < 0.05), with a statistically significant difference. The breastfeeding rate in the rooming-in with continuous SSC group was higher than that in the routine rooming-in care group (*P* < 0.05), showing a statistically significant difference. The use of jaundice medication in the rooming-in with continuous SSC group was lower than in the routine rooming-in care group (*P* < 0.05), with a statistically significant difference. Multivariate analysis of newborn weight loss greater than 7% revealed that cesarean delivery was a risk factor for excessive weight loss. Multivariate analysis of weight loss greater than 9% indicated that continuous SSC was a protective factor, while mixed feeding was a risk factor. Multivariate analysis of jaundice medication use showed that cesarean delivery and mixed feeding were risk factors, while previous deliveries and SSC were protective factors.

**Conclusion:**

Mother-infant rooming-in with continuous SSC promotes appropriate weight gain in newborns, increases breastfeeding rates, and reduces the need for jaundice medication.

## 1 Introduction

Skin-to-skin contact (SSC) refers to placing the newborn on the mother's exposed chest or abdomen, enabling direct, uncovered physical contact between the two without the use of clothes or blankets to separate the skin (1, 2). This care method, also known as Kangaroo Mother Care (KMC), promotes critical survival mechanisms in newborns through continuous SSC. It helps prevent early weight loss and hypoglycemia, while also facilitating breastfeeding. SSC is applicable in various settings, including delivery rooms, operating rooms, postpartum observation units, and maternity wards (3, 4).

Globally, SSC between mother and infant has been widely adopted in many countries. In 2013, the World Health Organization (WHO) introduced the Early Essential Newborn Care (EENC) guidelines, with continuous SSC as one of the core interventions. These guidelines recommend uninterrupted SSC day and night, only interrupted for diaper changes (5), and suggest that the practice should not be influenced by the newborn's gestational age, birth weight, or health status (6). The United Nations Children's Fund (UNICEF) also encourages immediate SSC after birth, maintaining it for at least one hour, with the option to continue beyond that, without specifying a set duration (7).

In Nordic countries such as Sweden and Norway, SSC has become a standard practice in neonatal care. Studies (1) have shown that SSC helps stabilize the newborn's body temperature, heart rate, and respiration, while improving breastfeeding success rates. In the United States, many hospitals have incorporated SSC into their standard care protocols. In China, the adoption of mother-infant SSC began later, but it has gradually gained attention in recent years. In 2016, China introduced the WHO's EENC guidelines, and with the support of WHO and UNICEF, relevant clinical practice recommendations were published (8, 9). These recommendations emphasized the importance of sustained SSC between mother and newborn.

However, the widespread implementation and promotion of SSC in China remains insufficient. Many hospitals have not yet adopted it as a routine care practice, and even in those that have, there are inconsistencies in operational protocols and maternal cooperation, which affect its effectiveness. Key barriers include limited healthcare resources in rural and underdeveloped regions, inadequate training of medical staff on SSC techniques, cultural perceptions prioritizing traditional postpartum care practices over modern evidence-based methods, and maternal anxiety about neonatal safety during prolonged skin contact. Given China's unique healthcare landscape where 32% of maternities still lack basic SSC equipment and 45% of healthcare providers report insufficient training in kangaroo care techniques, addressing these barriers is critical to improving neonatal outcomes nationwide (8, 9). This study aims to explore the effects of continuous mother-infant SSC in rooming-in care, focusing on its impact on newborn weight changes, breastfeeding success rates, and the incidence of neonatal jaundice. The goal is to verify its effectiveness and safety, providing reference data for the development and implementation of standardized SSC guidelines in China.

## 2 Materials and methods

### 2.1 General information

This study involved 2205 parturients and their full-term neonates, all of whom gave birth at the Shanghai Changning District Maternal and Child Health Care Hospital between January and December 2022. Inclusion criteria: women aged 18–40 years, singleton pregnancies, no pregnancy-related complications or comorbidities, and gestational age between 37 and 42 weeks. Exclusion criteria: preterm or post-term neonates, Apgar score ≤7, maternal fever (temperature ≥37.5°C) during delivery, voluntary withdrawal from follow-up, or communication difficulties. The study was approved by the Ethics Committee of Shanghai Changning District Maternal and Child Health Care Hospital.

### 2.2 Study methods

Both groups of neonates received healthcare measures within 90 min after birth, in accordance with the *Consensus on Basic Healthcare Techniques for Neonates in China (2017)* (8, 9). These measures included routine neonatal management: drying the body, SSC between the neonate and mother, delayed cord clamping, and umbilical care; early initiation and support for breastfeeding; routine treatments (vitamin K administration, eye care, vaccination, weighing, and clinical examination). Following these procedures, the neonates were transferred to mother-infant cohabitation rooms.

#### 2.2.1 Routine rooming-in care group

Routine neonatal care was implemented: (1) Breastfeeding: Encouragement and assistance for on-demand feeding, with breastfeeding status assessed before discharge. (2) Thermal protection and bathing: Room temperature maintained at 22–24°C, with bathing occurring 24 h post-birth. (3) Identification of risk signs: Neonates underwent a comprehensive physical examination, with particular attention to identifying any warning signs. (4) Discharge Guidance: Prior to discharge, neonates received a thorough examination, and parents were provided with counseling regarding signs that would require immediate medical attention. Parents were also instructed on neonatal healthcare services.

Neonates in the routine rooming-in care group received standardized care aligned with China's Clinical Implementation Recommendations for Early Essential Newborn Care Technologies (2017) (8), including: (1) Breastfeeding support: Nurses encouraged on-demand feeding and provided basic assistance (e.g., positioning guidance), but structured continuous SSC was not implemented beyond the initial 90-minute EENC protocol. (2) Kangaroo Mother Care (KMC) policy: Brief SSC was offered during the immediate postpartum period (<1 h), as recommended by national guidelines, but sustained daily SSC sessions were not routinely provided. (3) Lactation consultant services: Certified lactation consultants were available but provided individualized guidance only upon maternal request or when feeding difficulties (e.g., inadequate weight gain) were identified. (4) Formula supplementation: Formula milk was introduced according to institutional protocols if breastfeeding was deemed insufficient, and breast pumps were not universally available but reserved for specific cases (e.g., delayed lactation or preterm infants).

#### 2.2.2 Rooming-in with continuous SSC group

In addition to routine neonatal care, SSC care between mother and infant was implemented by the responsible nurse. All healthcare personnel involved in the care received specialized training on mother-infant SSC to ensure they could guide and support mothers in performing the contact correctly. The specific interventions were as follows: (1) Environmental and Temperature Settings: The room should be kept clean, with the temperature maintained between 22 and 24°C. A soft blanket should be used to cover the infant's back to ensure warmth. (2) Maternal Position: The mother should be in a semi-reclining position (30°–60°), supported by cushions under the back and elbows to maintain comfort and relaxation. If the mother cannot maintain a semi-reclining position, a supine position may be used, with the infant lying obliquely or transversely on the mother's chest. (3) Post-Cesarean Section Mothers: The infant should lie vertically on the mother's chest, with the legs closely aligned with the mother's upper abdomen, avoiding contact with the incision on the lower uterus. The mother may choose to cradle the infant with both hands or support the infant with one hand while protecting the incision with the other hand. (4) Contact Details: The mother's chest and abdomen should be exposed, without the need for special cleaning of the breasts and nipples. The infant should be undressed and placed between the mother's breasts, ensuring there are no obstacles between them. The infant's shoulders should make contact with the mother's chest, and the back should be kept warm with a blanket. (5) Supporting the Infant: The mother should support the infant's buttocks with both hands and use her arms to prevent the infant from sliding. The infant's head, torso, and limbs should be free to move. (6) Frequency and Duration of SSC: SSC should occur twice daily, once in the morning and once in the evening, with each session lasting 60 min. The total number of SSC sessions per infant averaged 6 sessions (2 sessions/day × 3 days) based on the standard 3-day postpartum hospital stay in this institution. The timing may be adjusted according to the mother's and infant's condition. It is recommended that the contact be performed when the infant is awake and the mother is not fatigued. (7) Education: Mothers should be informed about the infant's behaviors and responses, and encouraged to communicate with the infant when it is awake. If the infant does not show signs of rooting, its head should be turned to one side to ensure clear breathing. The mother should be comfortable and able to maintain the position for an extended period. The infant should be positioned close to the mother's chest, ensuring no obstruction to the airway for smooth breathing.

#### 2.2.3 Precautions

(1) Regular monitoring: The infant should be checked every hour within the first 24 h after birth, and every two hours thereafter. This ensures the safety of both mother and infant during SSC (the infant's nose and mouth should be visible and unobstructed).
(2) Regular recording of the infant's physiological parameters such as weight, temperature, heart rate, and respiration to assess the effectiveness of mother-infant SSC.
(3) When the infant falls asleep on the mother's chest, ensure that the infant's head is turned to one side. A 45°position is recommended to prevent the negative effects of a prone position on the infant's safety. If necessary, adjust the positions of the mother and infant. The infant's posture should be regularly checked and adjusted to prevent discomfort from maintaining a single position for too long.
(4) SSC should be suspended if the mother is using sedatives or analgesics or feels fatigued.
(5) Guide parents to recognize signs of distress in the infant, such as abnormal breathing or skin color, and instruct them to notify healthcare personnel immediately if these abnormalities are observed.

### 2.3 Observation indicators

The data for both the mothers and infants in both groups were collected by the responsible nurse.

(1) General information: Information including the mother's occupation, age, gestational age, education level, pregnancy risk level, mode of delivery, and infant's gender.
(2) Infant weight changes: Healthy, full-term newborns typically experience physiological weight loss after birth. The physiological weight loss is generally considered to range from 3% to 9%, with 7% being commonly used as the cutoff between normal and abnormal. In this study, the birth weight, the weight loss reaching 7% and 9% of the birth weight, and the number of infants in each group who experienced such weight loss were recorded and compared (8, 9).
(3) Breastfeeding status: According to medical instructions, the number of infants exclusively breastfed, mixed-fed, and formula-fed was recorded.
(4) Jaundice medication use: According to medical protocols, the number of newborns receiving treatment for jaundice during their hospital stay was recorded. Neonatal jaundice was diagnosed based on the following criteria: (1) Serum total bilirubin (TBil) ≥12 mg/dl in full-term infants; (2) TBil increase >5 mg/dl/day (86 μmol/L/day); (3) Clinical signs such as scleral icterus and skin jaundice. Treatment decisions were made based on: (1) TBil levels exceeding the phototherapy thresholds outlined in clinical guidelines; (2) Clinically significant hyperbilirubinemia with associated risk factors (e.g., isoimmune hemolytic disease, G6PD deficiency); (3) Failure of non-pharmacological interventions (e.g., increased breastfeeding frequency) (8). Medications used included oral Yinzhihuang Oral Liquid (0.5 ml/kg per dose, three times daily), administered according to institutional protocol for mild to moderate jaundice.

### 2.4 Statistical methods

All statistical analyses were performed using IBM SPSS Statistics for Windows, Version 27.0 (IBM Corp., Armonk, NY, USA). Data were analyzed according to variable types. Quantitative variables (e.g., birth weight, gestational age) were described as mean ± standard deviation (SD) and compared between groups using independent-samples *t*-tests. Categorical variables (e.g., feeding method, jaundice medication use) were presented as frequencies and percentages, and analyzed using Pearson's chi-square test (*χ*2) or Fisher's exact test when expected cell counts were <5. Multivariate logistic regression was applied to identify independent risk factors for excessive weight loss (>7% and >9% of birth weight) and jaundice medication use. Variables with *P* < 0.10 in univariate analyses were included in stepwise backward elimination models. Odds ratios (OR) with 95% confidence intervals (CI) were reported. Statistical significance was set at *α* = 0.05, with two-tailed *P* values < 0.05 considered significant.

## 3 Results

### 3.1 Comparison of baseline data

The baseline characteristics of the two groups were comparable. No significant differences were observed in maternal age, educational level, parity, gestational age, pregnancy risk level, neonatal gender, delivery mode, or prenatal fluid intake between the continuous SSC group and routine care group (all *P* > 0.05, Table 1). These findings confirmed the homogeneity of the study populations for subsequent analysis.

**Table 1:** Comparison of general information between the Two groups of study subjects [mean ± SD, *n* (%)].

Item	Rooming-in with continuous SSC group (*n* = 1,085)	Routine rooming-in care group (*n* = 1,120)	t/*χ*2	*P*
Age (years)	31.58 ± 3.65	31.68 ± 3.67	−0.641	0.521
Educational level		1.927	0.165	
Bachelor or below	978 (90.1%)	989 (88.3%)		
Master's or above	107 (9.9%)	131 (11.7%)		
First pregnancy		1.650	0.199	
Yes	678 (62.5%)	670 (59.8%)		
No	407 (37.5%)	450 (40.2%)		
First birth		0.717	0.397	
Yes	840 (77.4%)	850 (75.9%)		
No	245 (22.6%)	270 (24.1%)		
Gestational age (weeks)	39.24 ± 1.04	39.20 ± 1.02	0.912	0.362
Pregnancy risk level		1.989	0.158	
Low risk	974 (89.8)	1,025 (91.5%)		
High risk or above	111 (10.2)	95 (8.5%)		
Neonatal gender		0.207	0.649	
Male	563 (51.9%)	592 (52.9%)		
Female	522 (48.1%)	528 (47.1%)		
Delivery method		2.950	0.086	
Vaginal delivery	668 (61.6%)	650 (58.0%)		
Cesarean section	416 (38.4%)	470 (42.0%)		
Pre-delivery infusion volume (ml)	320.18 ± 76.32	315.36 ± 87.29	1.379	0.168

### 3.2 Comparison of weight loss in newborns between the two groups

This study analyzed the weight loss in newborns from both groups (Table 2). Statistical analysis indicated no significant difference in birth weight between the two groups (*P* > 0.05, rooming-in with continuous SSC group: 3348.72 ± 396.87 g, routine rooming-in care group: 3354.58 ± 390.96 g, *t* = −0.349, *P* = 0.727). Regarding the proportion of weight loss after birth, 7% of weight loss occurred in 40.0% (434/1,085) of the rooming-in with continuous SSC group and 5.6% (61/1,085) at 9%, while in the routine rooming-in care group, the respective proportions were 45.5% (511/1,120) and 10.5% (118/1,120). Chi-square tests showed that at 7% weight loss, *χ*2 = 7.120, *P* = 0.008; at 9%, *χ*2 = 17.839, *P* < 0.001. The rooming-in with continuous SSC group had a significantly lower proportion of weight loss compared to the routine rooming-in care group (*P* < 0.05), indicating a notable difference in weight loss between the two groups, which holds significant implications for neonatal care and health assessment.

**Table 2:** Comparison of weight loss after birth in newborns between the two groups [mean ± SD, *n* (%)].

Group	Birth weight (g)	Weight loss ≥7%	Weight loss ≥9%
Rooming-in with continuous SSC group (*n* = 1,085)	3,348.72 ± 396.87	434 (40.0%)	61 (5.6%)
Routine rooming-in care group (*n* = 1,120)	3,354.58 ± 390.96	511 (45.5%)	118 (10.5%)
t/χ2	−0.349	7.120	17.839
*p*	0.727	**0.008**	**<0.001**

Bold values indicate statistical significance.

### 3.3 Comparison of feeding practices between the two groups of newborns

This study compared the feeding practices of newborns in the rooming-in with continuous SSC group (*n* = 1085) and the routine rooming-in care group (*n* = 1120), as shown in Table 3. The exclusive breastfeeding rate in the rooming-in with continuous SSC group was 64.4%, significantly higher than the 39.2% in the routine rooming-in care group (*χ*2 = 140.443, *P* < 0.001), indicating that the rooming-in with continuous SSC group was more effective in promoting exclusive breastfeeding. Among non-exclusive breastfeeding, the proportion of breastfeeding combined with formula feeding in the rooming-in with continuous SSC group was 35.6%, lower than the 60.8% in the routine rooming-in care group. Regarding the introduction of formula at different stages, the rooming-in with continuous SSC group introduced formula before weight loss in 14.9%, after weight loss in 12.0%, and after recovery to normal weight in 8.7%, all of which were lower than the 25.3%, 22.1%, and 13.5% in the routine rooming-in care group, respectively, with statistically significant differences (*χ*2 values of 82.954 and 43.811, *P* < 0.001). In summary, the routine rooming-in care group had a higher proportion of formula introduction at each stage compared to the rooming-in with continuous SSC group, suggesting that the rooming-in with continuous SSC group's feeding strategy focused more on maintaining breastfeeding and reducing formula supplementation, whereas the routine rooming-in care group introduced formula earlier and in greater amounts.

**Table 3:** Comparison of infant feeding conditions between the two groups [n(%)].

Group	Exclusive Breastfeeding	Breast milk + Formula milk	Breast milk + Formula milk		
			Breast milk + Formula milk (Before weight loss)	Breast milk + Formula milk (After weight loss)	Breast milk + Formula milk (After weight recovery)
Rooming-in with continuous SSC group (*n* = 1,085)	699 (64.4%)	386 (35.6%)	162 (14.9%)	130 (12.0%)	94 (8.7%)
Routine rooming-in care group (*n* = 1,120)	439 (39.2%)	681 (60.8%)	283 (25.3%)	247 (22.1%)	151 (13.5%)
*χ* ^2^	140.443^a^	80.721^a^	82.954^a^	43.811^a^	
*p*	**<0.001**	**<0.001**	**<0.001**	**<0.001**	

Bold values indicate statistical significance.

^a^ Compared with the exclusive breastfeeding group.

### 3.4 Comparison of medication for neonatal jaundice between the two groups

This study compared the use of medication for neonatal jaundice in the rooming-in with continuous SSC group (*n* = 1085) and the routine rooming-in care group (*n* = 1120). In the rooming-in with continuous SSC group, 591 cases (54.5%) received medication, while 494 cases (45.5%) did not. In the routine rooming-in care group, 739 cases (66.0%) received medication, and 381 cases (34.0%) did not. Statistical analysis showed *χ*² = 30.514, *P* < 0.001, indicating that the proportion of medication use in the rooming-in with continuous SSC group was significantly lower than in the routine rooming-in care group, with a statistically significant difference (*P* < 0.05), as detailed in Table 4.

**Table 4:** Comparison of jaundice medication usage in newborns between the two groups [n (%)].

Group	Medicated cases	Non-medicated cases	*χ* ^2^	*p*
Rooming-in with continuous SSC group (*n* = 1,085)	591 (54.5%)	494 (45.5%)	30.514	**<0.001**
Routine rooming-in care group (*n* = 1,120)	739 (66.0%)	381 (34.0%)		

Bold value indicate statistical significance.

### 3.5 Multivariate analysis of infant weight loss greater than 7%

As shown in Table 5, a multivariate logistic stepwise regression analysis was performed using weight loss greater than 7% as the dependent variable, with factors including group, feeding method, maternal age, gestational age, educational level, pregnancy risk level, delivery method, and neonatal gender. The significant factor identified was delivery method, with cesarean section identified as a risk factor for infant weight loss greater than 7%.

**Table 5:** Univariate and multivariate logistic regression analysis of factors influencing infant weight loss >7%.

Variable	Univariate analysis					Multivariate analysis^a^				
	B	SE	Wald *χ*^2^	*P*	OR (95% CI)	B	SE	Wald *χ*^2^	*P*	OR (95% CI)
Rooming-in with continuous SSC group (vs. Routine rooming-in care group)	−0.288	0.302	0.909	0.340	0.750 (0.415–1.355)	–	–	–	–	–
Mode of delivery (vs. vaginal delivery)	0.384	0.106	13.124	<0.001	1.468 (1.193–1.807)	0.562	0.094	35.745	<0.001	1.754 (1.459–2.109)
Feeding method (vs. breastfeeding)	0.289	0.171	2.856	0.091	1.335 (0.955–1.867)	–	–	–	–	–
Maternal age	0.194	0.275	0.498	0.481	1.214 (0.708–2.081)	–	–	–	–	–
Education level (vs. undergraduate or below)	0.216	0.257	0.706	0.401	1.241 (0.750–2.054)	–	–	–	–	–
First pregnancy (vs. not first pregnancy)	0.118	0.402	0.086	0.769	1.125 (0.512–2.474)	–	–	–	–	–
Parity (vs. first child)	−0.301	0.168	3.210	0.073	0.740 (0.532–1.029)	–	–	–	–	–
Gestational age	0.420	0.247	2.891	0.089	1.522 (0.938–2.470)	–	–	–	–	–
Pregnancy risk level (vs. low risk)	0.333	0.208	2.563	0.109	1.395 (0.928–2.097)	–	–	–	–	–
Newborn sex (vs. male)	−0.283	0.299	0.896	0.344	0.754 (0.419–1.354)	–	–	–	–	–
Pre-delivery fluid intake	0.309	0.273	1.281	0.258	1.362 (0.798–2.326)	–	–	–	–	–

^a^ Multivariate logistic regression analysis was performed for all significant factors from the univariate logistic regression analysis.

### 3.6 Multivariate analysis of infant weight loss greater than 9%

As shown in Table 6, a multivariate logistic stepwise regression analysis was performed using weight loss greater than 9% as the dependent variable, with factors including group, feeding method, maternal age, gestational age, educational level, pregnancy risk level, delivery method, and neonatal gender. The final significant factors identified were delivery method, group, and feeding method. Cesarean section was identified as a risk factor for infant weight loss greater than 9%; the rooming-in with continuous SSC group was found to be a protective factor against weight loss greater than 9%; and mixed feeding was identified as a risk factor for weight loss greater than 9%.

**Table 6:** Univariate and multivariate logistic regression analysis of factors influencing infant weight loss >9%.

Variable	Univariate analysis					Multivariate analysis^a^				
	B	SE	Wald *χ*^2^	*P*	OR (95% CI)	B	SE	Wald *χ*^2^	*P*	OR (95% CI)
Maternal age	0.403	0.260	2.403	0.121	1.496 (0.899–2.491)	–	–	–	–	–
Education level (vs. undergraduate or below)	0.382	0.222	2.961	0.085	1.465 (0.948–2.264)	–	–	–	–	–
First pregnancy (vs. not first pregnancy)	0.198	0.374	0.280	0.597	1.219 (0.586–2.537)	–	–	–	–	–
Parity (vs. first child)	−0.206	0.453	0.207	0.649	0.814 (0.335–1.978)	–	–	–	–	–
Gestational age	−0.677	0.453	2.233	0.135	0.508 (0.209–1.235)	–	–	–	–	–
Pregnancy risk level (vs. low risk)	0.323	0.184	3.082	0.079	1.381 (0.963–1.981)	–	–	–	–	–
Newborn sex (vs. male)	0.143	0.304	0.221	0.638	1.154 (0.636–2.094)	–	–	–	–	–
Pre-delivery fluid intake	−0.283	0.212	1.782	0.182	0.754 (0.497–1.142)	–	–	–	–	–
Mode of delivery (vs. vaginal delivery)	0.429	0.148	8.404	0.004	1.536 (1.149–2.053)	0.586	0.167	11.086	<0.001	1.797 (1.273–2.537)
Rooming-in with continuous SSC group (vs. Routine rooming-in care group)	−0.834	0.273	9.333	0.002	0.434 (0.254–0.742)	−0.905	0.175	26.744	<0.001	0.405 (0.287–0.570)
Feeding method (vs. breastfeeding)	0.336	0.154	4.760	0.029	1.399 (1.035–1.892)	0.459	0.169	7.377	0.007	1.582 (1.136–2.204)

^a^ Multivariate logistic regression analysis was performed for all significant factors from the univariate logistic regression analysis.

### 3.7 Multivariate analysis of nutritional infants’ jaundice medication use

As shown in Table 7, a multivariate logistic stepwise regression analysis was performed using jaundice medication use in infants as the dependent variable, with factors including group, feeding method, maternal age, gestational age, educational level, pregnancy risk level, delivery method, neonatal gender, and parity. The final significant factors identified were delivery method, parity, group, and feeding method. Cesarean section was found to be a risk factor for jaundice medication use in infants; previous childbirth was identified as a protective factor; the rooming-in with continuous SSC group was found to be a protective factor; and mixed feeding was a risk factor for jaundice medication use in infants.

**Table 7:** Univariate and multivariate logistic regression analysis of factors influencing neonatal jaundice medication use.

Variable	Univariate analysis					Multivariate analysis^a^				
	B	SE	Wald *χ*^2^	*P*	OR (95% CI)	B	SE	Wald *χ*^2^	*P*	OR (95% CI)
Maternal age	0.392	0.257	2.327	0.127	1.480 (0.894–2.449)	–	–	–	–	–
Education level (vs. undergraduate or below)	0.505	0.637	0.628	0.428	1.657 (0.475–5.775)	–	–	–	–	–
First pregnancy (vs. not first pregnancy)	0.109	0.442	0.061	0.805	1.115 (0.469–2.652)	–	–	–	–	–
Parity (vs. first child)	−0.294	0.138	4.539	0.033	0.745 (0.569–0.977)	−0.382	0.139	7.553	0.006	0.682 (0.520–0.896)
Gestational age	−0.328	0.482	0.463	0.496	0.720 (0.280–1.853)	–	–	–	–	–
Pregnancy risk level (vs. low risk)	0.302	0.516	0.343	0.558	1.353 (0.492–3.719)	–	–	–	–	–
Newborn sex (vs. male)	−0.204	0.210	0.944	0.331	0.815 (0.540–1.231)	–	–	–	–	–
Pre-delivery fluid intake	−0.313	0.385	0.661	0.416	0.731 (0.344–1.555)	–	–	–	–	–
Mode of delivery (vs. vaginal delivery)	0.304	0.129	5.554	0.018	1.355 (1.052–1.745)	0.286	0.096	8.875	0.003	1.331 (1.103–1.607)
Rooming-in with continuous SSC group (vs. Routine rooming-in care group)	−0.482	0.192	6.302	0.012	0.618 (0.424–0.900)	−0.650	0.095	46.814	<0.001	0.522 (0.433–0.629)
Feeding method (vs. breastfeeding)	0.527	0.163	10.453	<0.001	1.694 (1.231–2.331)	0.779	0.108	52.027	<0.001	2.179 (1.764–2.693)

^a^ Multivariate logistic regression analysis was performed for all significant factors from the univariate logistic regression analysis.

## 4 Discussion

### 4.1 Continuous SSC and rooming-in reduce the incidence of excessive physiological weight loss in newborns

The results of this study indicate that continuous SSC and rooming-in significantly reduced the proportion of newborns experiencing marked physiological weight loss. SSC facilitates warmth, comfort, and emotional bonding between mother and infant, helping the newborn better adapt to the external environment, reducing energy expenditure, and thus mitigating the extent of weight loss. Additionally, SSC stimulates the newborn's rooting reflex, promoting earlier and more frequent breastfeeding, which ensures timely and adequate nutritional intake, further reducing the likelihood of weight loss. Consistent with our findings, several international studies support the notion that continuous SSC can decrease newborn weight loss. A study by Takahashi et al. (10) showed that extending SSC not only improved neonatal cardiopulmonary function but also reduced physiological weight loss by minimizing stress responses. Srivastava et al. (11) further emphasized that early implementation of SSC aids in promoting neonatal bowel movements and accelerates weight recovery. However, some studies suggest that the effect of SSC may be influenced by various factors. Brimdyr et al. (12) found that following cesarean delivery, the implementation rate of SSC was lower due to the mother's longer recovery time, which may diminish its positive impact on neonatal weight. Additionally, high-risk mothers or infants may have limited SSC due to physical conditions, thereby affecting its effectiveness.

Compared to international studies, research on continuous SSC in China is relatively scarce, with most focusing on its role in promoting breastfeeding. While the results of this study are generally consistent with international research, there are some differences, such as the actual implementation of SSC in different medical institutions in terms of duration and frequency. In some hospitals in China, limited human resources or insufficient awareness of skin-to-skin care have led to shorter implementation times, which may affect the impact of SSC on neonatal weight management (13). Future efforts should focus on enhancing standardized management of SSC implementation to maximize its positive role in newborn care.

### 4.2 Continuous SSC and rooming-in improve breastfeeding rates

The results of this study demonstrate that the rate of exclusive breastfeeding in the routine rooming-in care group was significantly lower than that in the rooming-in with continuous SSC group (*P* < 0.05). The incidence of formula supplementation before significant physiological weight loss, after significant physiological weight loss, and after the restoration of physiological weight in the newborns was lower in the rooming-in with continuous SSC group than in the routine rooming-in care group (*P* < 0.05). Continuous implementation of SSC and rooming-in has a positive impact on the physical and mental health of both mother and infant, aiding the newborn's adaptation to the external environment and stimulating maternal breast milk production, thus promoting early breastfeeding (14). International studies, such as that by Camacho et al. (15), indicate that the first feeding reflex in newborns typically occurs between 20 and 40 min postpartum. However, in most medical institutions in China, the typical SSC duration is only 30 min, which is relatively short. During prolonged SSC, if signs of feeding behavior are observed in the newborn, immediate oral stimulation can effectively stimulate the release of lactogenic hormones and uterine contractions in the mother's body, which enhances breastfeeding and promotes the newborn's weight gain and growth (16, 17). Furthermore, a Cochrane systematic review (18) suggests that initiating and maintaining SSC for a longer duration is more beneficial for the successful establishment of breastfeeding. Despite this, according to WHO survey data, 44.0% of newborns worldwide are exclusively breastfed for the first 6 months, whereas the corresponding rate in China is only 29.2% (19). Therefore, the promotion of early and continuous SSC and rooming-in in China is of significant importance for improving breastfeeding outcomes.

### 4.3 Continuous SSC and rooming-in reduce the use of medication for neonatal jaundice

The results of this study show that the incidence of medication use for neonatal jaundice was significantly lower in the rooming-in with continuous SSC group than in the routine rooming-in care group (*P* < 0.05). A study by Prameela et al. (20) indicated that for newborns in the first 6–48 h after birth, the primary focus of SSC should be on the abdomen and back. Touching these areas can trigger sensory responses in the newborn's skin, which are transmitted to the intestinal control center in the brainstem. This enhances the defecation mechanism, accelerates bowel movements, and facilitates the smooth passage of stools. It also improves bile acid metabolism in the blood, reduces the liver's reabsorption of bile acids, and decreases the reabsorption of bilirubin, thereby alleviating the severity of physiological jaundice. Chen et al. (21) has shown that early SSC can promote the early passage of meconium, and during breastfeeding, oral and lip friction effectively promotes neonatal intestinal motility. This leads to on-demand feeding and increased feeding frequency, which significantly aids in the clearance of meconium, reduces the reabsorption of bilirubin in the blood, and effectively lowers bilirubin levels, alleviating skin and mucosal jaundice symptoms until they completely resolve. A study by Li et al. (22) confirmed that through SSC and breastfeeding, the duration of physiological jaundice could be successfully shortened, significantly reducing the time for the resolution of symptoms.

This study contributes novel evidence to the field of neonatal care by demonstrating the efficacy of continuous SSC in a Chinese population, where implementation of SSC remains suboptimal. Our findings highlight three key advancements: (1) continuous SSC significantly reduced excessive neonatal weight loss (≥7%: 40.0% vs. 45.5%; ≥9%: 5.6% vs. 10.5%), with cesarean delivery identified as a risk factor for weight loss and SSC as a protective factor against severe weight loss (≥9%); (2) SSC improved exclusive breastfeeding rates (64.4% vs. 39.2%) and delayed formula supplementation across all stages of weight recovery; and (3) SSC reduced jaundice medication use (54.5% vs. 66.0%), with cesarean delivery and mixed feeding linked to increased treatment needs, while parity and SSC were protective. These results extend previous research by providing robust evidence for SSC's role in mitigating risks associated with cesarean delivery and mixed feeding—common challenges in Chinese maternity care. Clinically, this study underscores the importance of integrating SSC into routine practice to optimize neonatal outcomes, particularly in settings with high cesarean rates. Future efforts should focus on implementing standardized SSC protocols, addressing cultural barriers, and evaluating long-term benefits to inform national guidelines.

This study has several limitations inherent to its retrospective design. Selection bias may exist due to non-randomized grouping, and unmeasured confounding factors (e.g., maternal motivation for SSC) could influence results. Additionally, data on long-term outcomes (e.g., breastfeeding duration beyond hospital discharge) were not available.

## 5 Conclusion

This study highlights the pivotal role of continuous skin-to-skin contact (SSC) in improving neonatal outcomes within the Chinese healthcare system. To fully harness its benefits, future research should adopt longitudinal designs to assess long-term effects, including sustained breastfeeding rates and neurodevelopmental trajectories, while carefully controlling for confounding factors such as maternal motivation and cultural influences. Furthermore, randomized controlled trials are warranted to substantiate the protective effects of SSC against excessive weight loss and jaundice, particularly in high-risk populations such as cesarean-delivered infants. From a policy perspective, integrating standardized SSC protocols into routine clinical practice, alongside targeted training for healthcare professionals, is essential to mitigating implementation challenges, including inconsistent adherence and resource constraints. Public health initiatives should also prioritize parental education to enhance awareness of SSC benefits and address cultural preferences that may favor traditional postpartum care. By fostering interdisciplinary collaboration among healthcare institutions, policymakers, and communities, these strategies can strengthen evidence-based neonatal care and contribute to national efforts to improve maternal and child health outcomes.

## Data Availability

The original contributions presented in the study are included in the article/Supplementary Material, further inquiries can be directed to the corresponding authors.
